# A chromosome-scale assembly of the early-flowering *Prunus campanulata* and comparative genomics of cherries

**DOI:** 10.1038/s41597-023-02843-3

**Published:** 2023-12-21

**Authors:** Yuxi Hu, Chao Feng, Baohuan Wu, Ming Kang

**Affiliations:** 1 State Key Laboratory of Plant Diversity and Specialty Crops, South China Botanical Garden, Chinese Academy of Sciences, Guangzhou, 510650 China; 2 University of Chinese Academy of Sciences, Beijing, 100049 China; 3 South China National Botanical Garden, Guangzhou, 510650 China

**Keywords:** Comparative genomics, Plant evolution

## Abstract

*Prunus campanulata* is an important flowering cherry germplasm of high ornamental value. Given its early-flowering phenotypes, *P. campanulata* could be used for molecular breeding of ornamental species and fruit crops belonging to the subgenus *Cerasus*. Here, we report a chromosome-scale assembly of *P. campanulata* with a genome size of 282.6 Mb and a contig N50 length of 12.04 Mb. The genome contained 24,861 protein-coding genes, of which 24,749 genes (99.5%) were functionally annotated, and 148.20 Mb (52.4%) of the assembled sequences are repetitive sequences. A combination of genomic and population genomic analyses revealed a number of genes under positive selection or accelerated molecular evolution in *P. campanulata*. Our study provides a reliable genome resource, and lays a solid foundation for genetic improvement of flowering cherry germplasm.

## Background & Summary

The genus *Prunus* (family Rosaceae) contains many economically important plant species, such as peach, plum, apricot, almond, and cherry, grown for food and landscaping purposes. The subgenus *Cerasus* is classified within the genus *Prunus* with a corymbose inflorescence, comprises approximately 57 species of flowering trees or shrubs^1,2^. *Cerasus* has a worldwide distribution, with most species occurring mainly in the temperate zone of the northern hemisphere^3^. The subgenus *Cerasus* is believed to have originated in East Asia and then spread to West Asia^2^. A number of species in the subgenus *Cerasus* are economically and commercially important fruit crops, such as sweet cherry (*Prunus avium*), sour cherry (*Prunus cerasus*), and Chinese cherry (*Prunus pseudocerasus*), whose fruit can be either consumed raw or used for the production of jam or liquor^4^. Many *Cerasus* are of high ornamental value, owing to their graceful tree shape and attractive flowers, and are thus used for commercial and residential landscaping purposes.

Flowering cherries have been cultivated for over 1,000 years^5^. Centuries of propagation and cultivation of flowering cherries have produced a variety of natural and artificial hybrids, most of which are derived from crosses among 10 diploid species, including *P. apetala*, *P. campanulata*, *P. incisa*, *P. jamasakura*, *P. leveilleana*, *P. maximowiczii*, *P. nipponica*, *P. sargentii*, *P. spachiana* and *P. speciosa*^6,7^. Although most wild flowering cherries are distributed in China, modern flowering cherry cultivars are mainly derived from native Japanese taxa and their hybrids. Only two wild species native to China, *P. campanulata* and *P. pseudocerasus*, are believed to have contributed to modern cherry cultivars^5,8,9^.

*Prunus campanulata* (2n = 2x = 16), one of the main parents of flowering cherry cultivars, is considered as one of the four major ornamental cherry species, together with *P. yedoensis*, *P. subhirtella* var. *pendula*, and *P. cerasoides*^10^. *Prunus campanulata* is a typical early-flowering species, which usually blooms from January to March, and has a long flowering period (ca. 50 days). Thus, this species flowers much earlier and longer than *P. yedoensis* (April, 15–20 days) and *P. serrulata* (April–May, 11–14 days)^1^. Its attractive pink to magenta flowers and earlier blooming period make *P. campanulata* a popular choice for landscaping^11^. Unlike most *Cerasus* species, *P. campanulata* grows primarily in subtropical and tropical regions, showing adaptation to warmer climates. Therefore, *P. campanulata* possesses some desirable traits, such as early and prolonged flowering, anti-pollution effect, and heat tolerance, which could be used for breeding flowering cherry cultivars^12,13^. However, the lack of genome sequence information hinders our understanding of the mechanisms underlying heat tolerance and early flowering in *P. campanulata*.

**Fig. 1:**
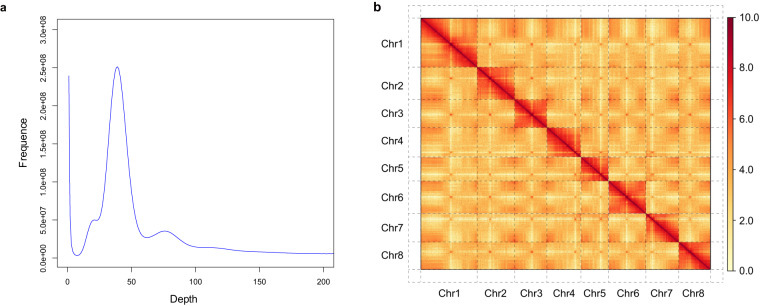
*K*-mer frequency distribution curve (**a**) and the interaction heat map (**b**) of the *Prunus campanulata* genome.

Here, we report a chromosome-level genome assembly of *P. campanulata*. PacBio HiFi reads (~97 × coverage) were used to assemble the genome yielding a contig assembly of ~282.6 Mb, with contig N50 value of 12.04 Mb (Table 1). The assembled contig size was close to the estimated genome size of 282.8 Mb based on *k*-mer estimates (Fig. 1a). With the aid of Hi-C sequencing (~176 × coverage) technologies, 92.3% of the contigs were anchored and oriented onto eight pseudomolecules, with a scaffold N50 length of 30.65 Mb (Fig. 1b, Table 1). We traced the evolutionary dynamics of genomes and gene families for *P. campanulata*. Applying comparative and evolutionary genomics approaches, we identified a number of genes that underwent positive selection or accelerated molecular evolution in *P. campanulata*. Among them, five candidate genes (*VIL1*, *PUB14*, *FD, DDL* and *SR45A*) have previously been demonstrated to be involved in the regulation of flowering time in other species, suggesting their potential association with the early-flowering traits of *P. campanulata*. Our results provide genetic resources for the genetic improvement and optimization of ornamentally and agriculturally important *Cerasus* species.

**Table 1:** Summary statistics of genome assembly and annotation for *Prunus campanulata*.

Assembly parameters	*Prunus campanulata*
**Assembly feature**	
Estimated genome size (Mb)	282.84
Total length of scaffolds (Mb)	282.64
N50 of scaffolds (Mb)	30.65
Total length of contigs (Mb)	282.63
N50 of contigs (Mb)	12.04
Mapping rate of reads from short-insert libraries (%)	99.25
CEGMA evaluation (%)	95.56
BUSCO evaluation (%)	99.10
**Genome annotation**	
Percentage of TE (%)	52.42
Percentage of LTRs (%)	31.91
No. of predicted protein-coding genes	24,861
No. of genes annotated to public database	24,749

## Methods

### Library construction and genome sequencing

For whole-genome sequencing, fresh young leaves were collected from a mature plant of *P. campanulata* grown at South China Agricultural University (Guangzhou, China) (23.1557° N, 113.3537° E). Genomic DNA was extracted from leaf tissue using a modified CTAB method^14^. Short-read sequencing libraries with an insert size of 350 bp were constructed and used for paired-end (PE) 150 bp sequencing on the Illumina NovaSeq 6000 platform. Reads with adapters, with > 10% unidentified nucleotides (N), and paired reads with more than 20% of base quality ≤ 5 in either paired read were filtered out. A total of 27.13 Gb of clean data was produced and used for the genome survey. For PacBio SMRT sequencing, the PacBio Sequel II platform was first used to generate sub-reads, and the sub-reads were then filtered by the ccs software using the parameter “min-passes = 3, min-rq = 0.99” to obtain 27.50 Gb of HiFi reads. A Hi-C library was constructed by chromatin crosslinking, restriction enzyme digestion (DpnII), end filling and biotin labeling, DNA purification and shearing, and extraction of biotin-containing fragments after sonication interruption. The Hi-C sequencing library was sequenced on Illumina PE150. The resulting sequencing data were filtered using the same filtering criteria as the short reads, retaining 49.75 Gb of clean data.

Five tissues including leaves, branches, flowers, fruits and roots were collected from the same *P. campanulata* tree for transcriptome sequencing. RNA-seq libraries were prepared and then subjected to PE150 sequencing on the Illumina NovaSeq 6000 platform.

### Genome size estimation, genome assembly and quality assessments

To estimate the genome size, heterozygosity and repeat content of *P. campanulata*, we performed *k*-mer frequency analysis based on the 17 *k*-mers depth distribution with GCE^15^ using Illumina short reads. Based on the *k*-mer analysis, the size of the *P. campanulata* genome was estimated to be ~282.8 Mb, with heterozygosity of 0.61% and repeat content of 47.7%.

**Fig. 2:**
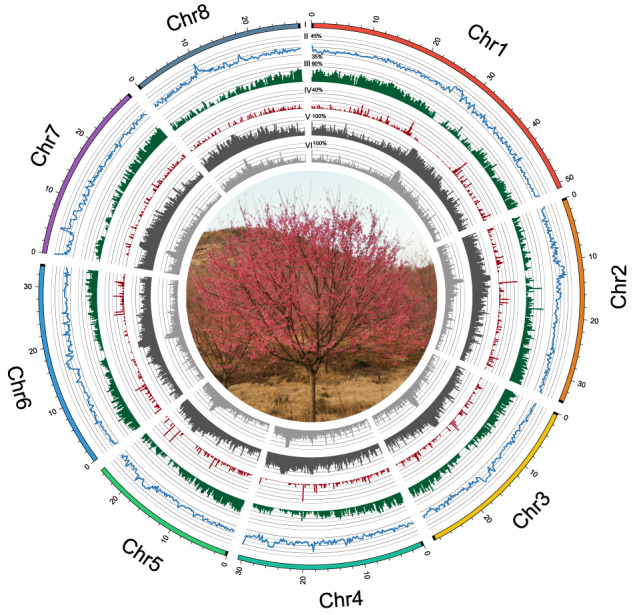
The genomic features of *P. campanulata*. Tracks from the outermost to the innermost circle separately represent the assembled eight pseudochromosomes (I), guanine-cytosine (GC) content (II), gene density (III), tandem duplication (IV), transposable element density (V), and long-terminal repeat density (VI). Black squares at the end of each chromosome in (I) represent telomeres. Photo credit: Ganbiao Xian.

The PacBio HiFi reads were assembled into the initial set of contigs using hifiasm v0.8^16^ with default parameters. The contig assembly had a total size of ~282.6 Mb, with a contig N50 value of 12.04 Mb. Genome completeness was assessed using the Benchmarking Universal Single-Copy Orthologs (BUSCO v4.1.2)^17^ program with the embryophyta_odb10 database and the Core Eukaryotic Genes Mapping Approach (CEGMA v2.5)^18^, which yielded 99.1% of the complete BUSCO genes and 95.6% of the core eukaryotic genes (Table 1). In addition, the filtered short reads were mapped against the assembled genome using the BWA-MEM v0.7.8^19^ algorithm to assess the accuracy of the assembly, and the mapping rate and coverage of the Illumina short reads were 99.02% and 99.92%, respectively. To achieve chromosome-level assembly, the ALLHiC algorithm^20^ was used to group, adjust the order and orientation of contigs and anchor the assembled contigs into eight pseudomolecules based on Hi-C data. After ALLHiC scaffolding, Hi-C interaction heat map was constructed using HiC-Pro v3.1.0^21^ and visualized using HiCPlotter^22^. Finally, a total of eight pseudomolecules were obtained, which contained 92.3% of the contigs. Telomere sequences (CCCTAAA/TTTAGGG repeats) were identified by searching the chromosome-level assembly using Telomere Identification toolKit (tidk, https://github.com/tolkit/telomeric-identifier). These repeat arrays were identified at both distal ends of pseudomolecules 2, 3, and 8, and at one distal end of pseudomolecules 1, 4, 5, 6, and 7 (Fig. 2). To evaluate the assembly continuity, the long terminal repeat (LTR) assembly index (LAI) value was employed using LTR_retriever^23^ by estimating the percentage of intact LTR elements. The LAI value of the genome assembly was 19.3, which almost reached the “gold standard” (LAI value > 20) of genome assembly proposed by Ou *et al*.^24^. Collectively, these results indicate a high quality of the *P. campanulata* genome assembly, thus ensuring the reliability of our subsequent analyses.

### Genome annotations

For repeat sequence annotation, we used a combined strategy of homology-based search and *de novo* prediction. The homology-based search was based on the Repbase database using RepeatMasker (http://www.repeatmasker.org/) and RepeatProteinMask (http://www.repeatmasker.org/) to search for interspersed repeat elements. The *de novo* prediction was based on a species-specific repeat database generated by LTR_FINDER^25^, Piler (http://www.drive5.com/piler/), RepeatScout (http://www.repeatmasker.org/) and RepeatModeler (http://www.repeatmasker.org/RepeatModeler.html). Using this library, we identified *de novo* involved repeats with RepeatMasker and predicted tandem repeats with TRF (http://tandem.bu.edu/trf/trf.html). Repetitive sequence annotation detected approximately 148.2 Mb of repetitive sequences in the *P. campanulata* genome, accounting for 52.4% of the total genome size (Table 2). In addition, we used RepeatMasker to mask the repetitive sequences as input for gene structure prediction.

**Table 2:** Summary of repeat contents in *P. campanulata*.

Type	DNA level		Protein level		Combined	
	(RepeatMasker)		(RepeatProteinMask)			
	Size (bp)	Percentage (%)	Size (bp)	Percentage (%)	Size (bp)	Percentage (%)
DNA Transposons	42,799,194	15.14	7,357,176	2.60	45,385,059	16.06
LINES	4,571,428	1.62	2,142,401	0.76	5,743,667	2.03
SINES	260,124	0.09	0	0.00	260,124	0.09
LTR	89,481,641	31.66	18,675,217	6.61	90,175,552	31.91
Satellites	1,221,740	0.43	0	0.00	1,221,740	0.43
Simple_repeats	2,697,511	0.95	0	0.00	2,697,511	0.95
Unknown	11,654,999	4.12	0	0.00	11,654,999	4.12
Total	146,116,181	51.70	28,113,983	9.95	148,147,077	52.42

For protein-coding gene structure prediction, we used a comprehensive approach that integrates homology-based prediction, *de novo* prediction, and RNA-Seq-based prediction. For homology-based prediction, Blast^26^ and Genewise^27^ were used to align the amino acid sequences from the *Malus domestica*, *P. serrulata*, *Vitis vinifera*, *A. thaliana*, and *P. salicina* genomes to the assembled *P. campanulata* sequences. Augustus^28^, GlimmerHMM^29^, SNAP^30^, GeneID^31^, and GenScan^32^ were used to predict de novo gene models. Cufflinks^33^ and PASA^34^ were applied to predict the gene models in the RNA-Seq-based prediction study. The results of the above three approaches were further integrated to generate a final non-redundant gene model set using EVidenceModeler^35^ and modified using PASA. We predicted 24,861 protein-coding gene models from the repeat-masked *P. campanulata* genome, of which 24,749 were functionally annotated against public databases (Table 3, Table 4). The distribution of genes and transposable elements (TEs) along each chromosome revealed that genes are more densely packed at the distal ends of chromosomes, whereas TEs are clustered around the centromeric regions of chromosomes (Fig. 2), thus following the typical distribution of monocentric plant genomes^36^. To annotate the putative function of the genes, we used BLASTP to perform homologous alignments against several public databases, including SwissProt, TrEMBL, the non-redundant protein database of NCBI (NR), GO, and KEGG^37^. In addition, we used InterProScan to predict gene motifs and domains, and obtained gene ontologies from InterPro^38^. For the annotation of non-coding RNAs, tRNAscan-SE (http://lowelab.ucsc.edu/tRNAscan-SE/) was employed to identify transfer RNAs (tRNAs). Ribosomal RNAs (rRNAs) were identified using BLASTN with an E-value of 1e-5 against rRNA sequences from related species. MicroRNAs (miRNAs) and small nuclear RNAs (snRNAs) were predicted using INFERNAL (http://infernal.janelia.org/) and searched against the Rfam database.

**Table 3:** Summary of predicted protein-coding genes in *P. campanulata*.

Approach	Software/species	Number	Average gene length (bp)	Average CDS length per gene(bp)	Average exons per gene	Average exon length (bp)	Average intron length (bp)
*De novo*	Augustus	26,465	2,411.79	1,111.79	1,111.79	257.89	392.62
	GlimmerHMM	41,528	5,139.14	727.59	727.59	257.53	2,416.99
	SNAP	35,394	3,845.62	737.17	737.17	204.91	1,196.70
	Geneid	31,387	3,804.80	957.03	957.03	212.45	812.56
	Genscan	20,821	8,384.80	1,425.22	1,425.22	212.31	1,218.20
Homolog	*Malus domestica*	25,812	3,059.67	1,066.69	4.05	263.48	653.75
	*Cerasus serrulata*	32,363	2,373.10	1,049.06	4.01	261.62	439.88
	*Vitis vinifera*	21,391	2,630.94	1,123.75	4.34	259.20	451.87
	*Arabidopsis thaliana*	24,423	2,263.16	1,067.89	3.84	278.21	421.11
	*Prunus salicina*	32,443	2,083.57	1,000.06	3.81	262.51	385.64
	*Prunus yedoensis*	35,622	1,795.22	806.52	3.51	229.91	394.23
RNA-Seq	Cufflinks	53,655	6,750.99	2,297.48	6.53	351.96	805.68
	PASA	62,247	2,799.00	1,073.49	4.86	220.81	446.85
EVM		29,352	2,406.14	1,081.29	4.35	248.85	396.04
PASA-update		29,189	2,419.83	1,100.36	4.38	250.96	389.84
**Final set**		**24,861**	**2,639.37**	**1,180.73**	**4.79**	**246.40**	**384.67**

**Table 4:** Summary of protein-coding gene annotation of *P. campanulata*.

Database	Number	Percentage (%)
Swissprot	18,093	72.8
NR	23,424	94.2
GO	22,229	89.4
KEGG	16,335	65.7
Pfam	16,557	66.6
InterPro	24,584	98.9
Annotated	24,749	99.5
Total	24,861	—

### Comparative genomic analysis across *Cerasus* species

**Fig. 3:**
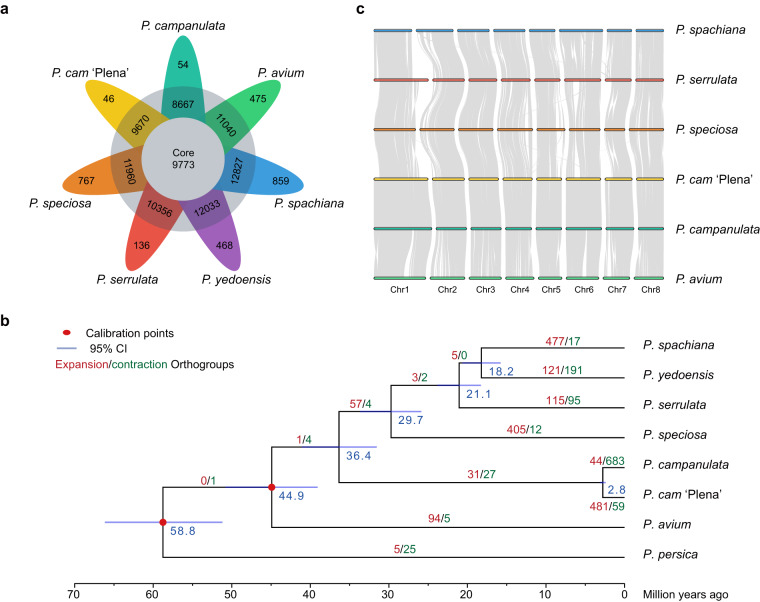
The evolutinary analysis of *Cerasus* genomes. (**a**) Flower plot showing the number of core orthogroups (center), orthogroups in a subset of species (annulus), and species-specific orthogroups (petals) for *Cerasus* species. (**b**) Estimation of the phylogeny, divergence times, and gene families of seven *Prunus* species. (**c**) Collinearity patterns among Cerasus species assembled to chromosomes.

We collected the protein sequences of the longest transcripts from *P. campanulata*, the double-flower cultivar *P. campanulat*a ‘Plena’^39^ and four other species of *Cerasus*, including *P. avium*^13^, *P. serrulata*^40^, *P. yedoensis*^41^, and *Cerasus* × *yedoensis*^7^. *Cerasus* × *yedoensis* was proposed to be an interspecific hybrid of *P. spachiana* and *P. speciosa*, and Shirasawa *et al*.^7^ assembled two haplotype phased genome sequences of this interspecific hybrid with SMRT sequencing. In this study, we used these two parental genome sequences to represent the genome assembly of *P. spachiana* and *P. speciosa*, respectively. We classify orhologues and orthogroups from the above six species using OrthoFinder v2.5.4^42^ with the parameter ‘-S diamond’. Based on the orthogroup clustering of six *Cerasus* species, we identified a total of 31,421 orthogroups, of which 9,773 were core orthogroups shared by all seven species, and 2,791 were single-copy orthogroups with only one gene per species (Fig. 3a). *Prunus campanulata* had 54 species-specific orthogroups, containing a total of 977 genes. Gene Ontology (GO) enrichment analysis performed using the R package topGO v2.38.1^43^ revealed that these genes were mainly involved in signaling processes such as regulation of transport, cell communication, and photoperiodism.

To construct the phylogenetic tree of *P. campanulata* and representative *Cerasus* species, we first performed orthogroup classification using the above-mentioned seven *Cerasus* species and *P. persica*^44^, and obtained 2,685 single-copy genes. Then, we used the parallel Alignment and back-Translation (paraAT) tool^45^ to align the protein sequences of 2,685 single-copy orthologous genes in parallel, and back-translated the aligned sequences into the corresponding aligned coding sequences (CDS). We constructed the maximum-likelihood (ML) phylogenetic tree for each gene using IQ-TREE v1.6.12^46^ with the parameter “-bb 1000”, setting *P. persica* as the outgroup. We then inferred the final species tree by summarizing a set of gene trees using ASTRAL-II v5.7.7^47^. We conducted the divergence time estimation using Bayesian Evolutionary Analysis Sampling Trees (BEAST) v2.6.6^48^, with two reported divergence times set as secondary calibrations: the ancestral node of *P. persica* and the subgenus *Cerasus* (mean: 58.2 MYA, Std dev: 4.3 MYA); the divergence of *P. avium* and *P. serrulata* (mean: 48.8 MYA, Std dev: 6.0 MYA)^2^. Markov chain Monte Carlo was run for 100,000,000 generations with 1,000 steps. Based on the ultrametric species tree and the results of the gene family clustering analysis, we used CAFE v4.2.1^49^ to identify the patterns of gene family evolution. We filtered out gene families with abnormal gene copy numbers, that is, those gene families containing more than 100 gene copy numbers in one or multiple species. The orthologous gene family expansions or contractions at each branch were considered significant at *p* < 0.01, and 44 gene families underwent significant expansion (*p* < 0.01), while 683 gene families underwent contraction during the evolution of *P. campanulata* (Fig. 3b). GO enrichment analysis suggested that genes in the expanded gene families were significantly enriched in oxidation-reduction processes involved in cellular respiration, such as oxidative phosphorylation and respiratory electron transport chain. Furthermore, we used MCscan (https://github.com/tanghaibao/jcvi/wiki/MCscan-(Python version) to measure collinearity between the six chromosome-level genomes of *Cerasus* species, which showed an overall high degree of collinearity, indicating that species within the subgenus *Cerasus* exhibit relatively conserved genomic synteny (Fig. 3c).

### Identification of positively selected genes and rapidly evolving genes

To search for the genomic footprint of natural selection that may be involved in flowering time regulation, we performed positive selection analysis and rapidly evolving gene analysis using 2,893 single-copy orthologous genes from *P. campanulata* and five late-flowering *Cerasus* species. For the positive selection analysis, we used the branch-site model of the codeml program in the PAML v4.9 package^50^, which allows for differential selective pressure both among amino acid sites and between branches on the tree. We then performed a likelihood ratio test (LRT) to compare the likelihood differences between the two models used, modelA, which allows sites to be positively selected on the foreground branch (*P. campanulata*), and the null model, in which sites could have evolved neutrally and/or under purifying selection. Genes with a Chi-squared test *p* value < 0.05 were considered to be positively selected in *P. campanulata*.

To identify rapidly evolving genes, we used the branch model incorporated in the PAML v4.9 package, which assumes that genes evolve at an accelerated rate on the foreground branch. Similarly, we compared the null model (model = 0) and the alternative model (model = 2) using LRT, and genes with *p* value < 0.05 and a higher ω value for the foreground branch than the background branch were considered to be evolving significantly faster in *P. campanulata*.

## Data Records

The raw data of Illumina PE150 reads, PacBio HiFi reads and Hi-C reads described in this manuscript have been submitted to the National Center for Biotechnology Information (NCBI) with accession number SRR22071520^51^, SRR26446899^52^, SRR25019708^53^ under BioProject accession number PRJNA895162. The RNA-seq data for different tissues are also under PRJNA895162. The genome assembly has been deposited at GenBank under the accession JAXCME000000000^54^. The genome assembly and annotation of *P. campanulata* have been also submitted to the Genome Database for Rosaceae (GDR; www.rosaceae.org) under the accession number tfGDR1074^55^. The protein sequences used in the comparative genomic analyses and the output files including orthogroup clustering analysis and PAML analysis are available at Figshare with the 10.6084/m9.figshare.23694168^56^.

## Technical Validation

### Evaluating the quality of the genome assembly

We evaluated the quality of our assembly using three methods: (1) Performing BUSCO and CEGMA analyses. The BUSCO analysis revealed 99.10% complete BUSCO genes, of which 96.30% were single-copy genes. In addition, 0.30% of BUSCO genes were fragmented, and 0.60% were missing from the genome. The CEGMA analysis detected 95.6% of the eukaryotic core genes in the assembly; (2) Calculation of the paired-end read coverage and mapping rate by mapping the short Illumina reads to our genome assembly, which showed 99.02% genome coverage and 99.92% mapping rate; (3) Calculation of the LAI value. The LAI value was 19.3. These results confirmed the high quality of our assembly.

### Evolution of genes in *P. campanulata*

**Fig. 4:**
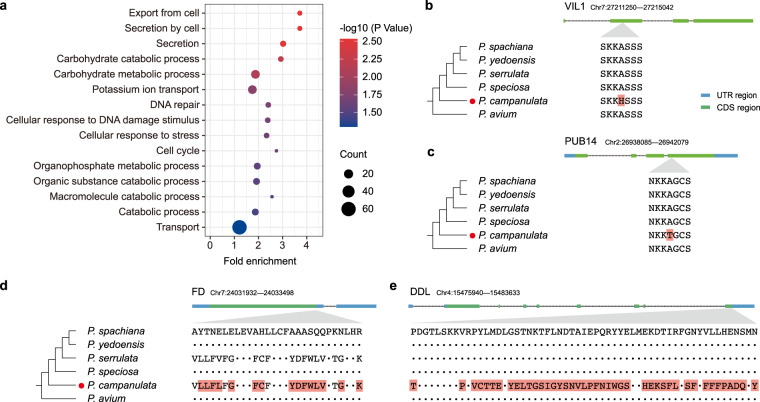
Evolution of PSGs in *P. campanulata*. (**a**) GO enrichment analysis of positively selected genes in the *P. campanulata* genome. (**b**–**e**) Alignment of deduced amino acid sequences of four positively selected genes (PSGs), *VIL1*, *PUB14*, *FD* and *DDL*. Sites marked in red were potentially under positive selection in *P. campanulata* according to the empirical Bayesian approach. Dots represent sequence identity with the *P. spachiana* genome.

Of the 2,893 single-copy orthologs, 248 genes were considered to be positively selected in *P. campanulata* with the chi-squared test *p* value < 0.05. Functional enrichment analysis of the positively selected genes (PSGs) revealed that they showed enrichment in some potentially resistance-related GO terms, such as potassium ion transport, DNA repair, cellular response to DNA damage stimulus, and cellular response to stress (Fig. 4a). Among the 248 PSGs, four genes (*VIL1*, *FD*, *PUB14* and *DDL*) were previously shown to be associated with flowering time regulation in other species (Fig. 4b–e)^57–60^.

We identified a total of 94 rapidly evolving genes (REGs), that showed a higher ω value in *P. campanulata* than in the background. GO enrichment analysis of these REGs in *P. campanulata* revealed that they were significantly enriched in monovalent inorganic cation transport (GO:0015672) and potassium ion transport (GO:0006813). One of the REGs, *SR45A*, encoding the serine/arginine-rich (SR) splicing factor SR45a, was shown to be associated with the regulation of flowering date in sweet cherry^61^. Our evolutionary analysis of these genes in *P. campanulata* provides new evidence for loci associated with flowering time regulation and provides prime candidates for future breeding of crops such as sweet cherry.

## Data Availability

All software used in this study was run according to the official instructions. The version and parameters of the software and the other custom codes used were described in Methods. Anything not specified in Methods was run with default parameters.
